# Phase II study of two dose schedules of C.E.R.A. (Continuous Erythropoietin Receptor Activator) in anemic patients with advanced non-small cell lung cancer (NSCLC) receiving chemotherapy

**DOI:** 10.1186/1745-6215-8-8

**Published:** 2007-03-06

**Authors:** Vera Hirsh, John Glaspy, Paul Mainwaring, Christian Manegold, Rodryg Ramlau, Joseph E Eid

**Affiliations:** 1 Royal Victoria Hospital, McGill University Health Center, Montreal, Quebec, Canada; 2 Division of Hematology-Oncology, UCLA School of Medicine, Los Angeles, CA, USA; 3 Medical Oncology, Mater Adult Hospital, South Brisbane, Australia; 4 Heidelberg University Medical Center, Mannheim, Germany; 5 Regional Center of Lung Diseases, Poznań, Poland; 6 Hoffmann-La Roche Inc., Nutley, NJ, USA

## Abstract

**Background:**

C.E.R.A. (Continuous Erythropoietin Receptor Activator) is an innovative agent with unique erythropoietin receptor activity and prolonged half-life. This study evaluated C.E.R.A. once weekly (QW) or once every 3 weeks (Q3W) in patients with anemia and advanced non-small cell lung cancer (NSCLC) receiving chemotherapy.

**Methods:**

In this Phase II, randomized, open-label, multicenter, dose-finding study, patients (n = 218) with Stage IIIB or IV NSCLC and hemoglobin (Hb) ≤ 11 g/dL were randomized to one of six treatment groups of C.E.R.A. administered subcutaneously for 12 weeks: 0.7, 1.4, or 2.1 μg/kg QW or 2.1, 4.2, or 6.3 μg/kg Q3W. Primary endpoint was average Hb level between baseline and end of initial treatment (defined as last Hb measurement before dose reduction or transfusion, or the value at week 13). Hematopoietic response (Hb increase ≥ 2 g/dL or achievement of Hb ≥ 12 g/dL with no blood transfusion in the previous 28 days determined in two consecutive measurements within a 10-day interval) was also measured.

**Results:**

Dose-dependent Hb increases were observed, although the magnitude of increase was moderate. Hematopoietic response rate was also dose dependent, achieved by 51% and 62% of patients in the 4.2 and 6.3 μg/kg Q3W groups, and 63% of the 2.1 μg/kg QW group. In the Q3W group, the proportion of early responders (defined as ≥ 1 g/dL increase in Hb from baseline during the first 22 days) increased with increasing C.E.R.A. dose, reaching 41% with the highest dose. In the 6.3 μg/kg Q3W group, 15% of patients received blood transfusion. There was an inclination for higher mean Hb increases and lower transfusion use in the Q3W groups than in the QW groups. C.E.R.A. was generally well tolerated.

**Conclusion:**

C.E.R.A. administered QW or Q3W showed clinical activity and safety in patients with NSCLC. There were dose-dependent increases in Hb responses. C.E.R.A. appeared to be more effective when the same dose over time was given Q3W than QW, with a suggestion that C.E.R.A. 6.3 μg/kg Q3W provided best efficacy in this study. However, further dose-finding studies using higher doses are required to determine the optimal C.E.R.A. dose regimen in cancer patients receiving chemotherapy.

## Background

Non-small cell lung cancer (NSCLC) is one of the most common tumors diagnosed in Western countries. The predicted incidence of lung cancer in the US was 173,000 new cases in 2005 [1], of which most (over 80%) will have a diagnosis of NSCLC. The majority of patients with Stage IIIB (pleural effusion) or Stage IV NSCLC require chemotherapy as part of their management program, which is often toxic; current first-line standards of care for Stage III-IV NSCLC use platinum compounds (such as carboplatin or cisplatin) combined with gemcitabine, vinorelbine, or taxanes, usually administered in 3-week cycles [2-5].

The prevalence of anemia (defined as hemoglobin [Hb] < 11 g/dL) in patients with lung cancer ranges from 50% to 60%, and blood transfusions are common, with 30% to 40% of patients requiring this treatment [6,7]. The cause of cancer-related anemia is often multifactorial. It is associated with the excessive release of cytokines such as interleukin-1, tumor necrosis factor, and interferons, which interfere with the production of endogenous erythropoietin and inhibit erythroid bone marrow production [8,9]. The anemia is also exacerbated by the use of myelosuppressive combination chemotherapy, particularly with platinum compounds [5].

Anemia has a major impact on the quality of life (QoL) of patients with cancer, its symptoms including fatigue, dizziness, headache, and shortness of breath [10-12]. In patients with lung cancer, these symptoms are often enhanced by diminished pulmonary function and the frequent presence of comorbidities [11]. Anemia has also been shown to be associated with a diminished response to chemotherapy and a decreased survival in patients with NSCLC [13-15]. Blood transfusions provide effective acute relief of anemia, but their effects are short-lived and they are associated with significant risks such as transfusion reactions and transmission of infection [16,17].

Treatment of chemotherapy-associated anemia with the erythropoiesis-stimulating agents (ESAs), epoetin alfa, epoetin beta, and darbepoetin alfa, has been shown to increase Hb levels, thereby reducing the need for transfusions and improving QoL [18-22]. In anemic patients with cancer, ESAs were initially administered three-times weekly, a schedule that had already proved effective in patients with renal anemia. Recently, there have been moves towards once weekly (QW) administration with all ESAs [20,23,24]. Darbepoetin alfa has also been licensed recently for once every 3 weeks (Q3W) use in anemic patients receiving chemotherapy for cancer. Given the increased convenience of once per cycle dosing, it is important that new agents being developed for the treatment of chemotherapy-induced anemia are studied at intervals of Q3W.

Continuous Erythropoietin Receptor Activator (C.E.R.A.) is an innovative agent with unique receptor activity and a prolonged half-life. It is a chemically synthesized C.E.R.A. that differs from erythropoietin through the integration of amide bonds between amino groups and methoxy polyethylene glycolsuccinimidyl butanoic acid [25,26]. C.E.R.A. is currently in development to provide correction of anemia and stable control of Hb levels at extended administration intervals in patients with cancer [26,27].

Studies in healthy volunteers demonstrated that C.E.R.A. had lower systemic clearance and an increased elimination half-life (t_1/2_) compared with other ESAs, and superior potency *in vivo *with respect to the magnitude and duration of response [28,29]. Further healthy volunteer studies demonstrated rapid, dose-dependent increases in reticulocytes following either intravenous or subcutaneous (SC) administration of C.E.R.A. [30]. An exploratory Phase I–II dose-escalation study in anemic patients with multiple myeloma receiving myelosuppressive chemotherapy (selected as a more responsive population to ESA treatment based on their baseline ratio of observed/predicted erythropoietin levels) confirmed the long half-life of C.E.R.A. (median 6.3–9.7 days) [27]. Also, a dose-dependent increase in Hb response was observed with C.E.R.A. doses up to 8 μg/kg when administered Q3W by SC injection. An Hb increase of ≥ 2 g/dL in the first 6 weeks was achieved in 50.0%–62.5% of patients treated with C.E.R.A. 3.5–8.0 μg/kg Q3W.

This multicenter, open-label, randomized study was designed to evaluate the efficacy, safety, and pharmacokinetic profile of C.E.R.A. administered SC QW or Q3W to patients with anemia and advanced (Stage IIIB or IV) NSCLC receiving chemotherapy. A further aim was to determine the optimum C.E.R.A. dose. The dose range chosen was based on that used in the previous study in multiple myeloma patients [27].

## Methods

### Patients

Patients eligible for study inclusion were adults (aged ≥ 18 years) with Stage IIIB or IV NSCLC and a Hb level of ≤ 11 g/dL at screening. Patients were receiving first or second line chemotherapy at screening, which continued for at least 9 weeks during the study. Additionally, patients had an Eastern Cooperative Oncology Group (ECOG) performance status grade of 0–2 (first-line chemotherapy) or 0–1 (second-line chemotherapy) and a life expectancy of > 6 months.

Patients were excluded from the study if they had received a red blood cell transfusion or radiation therapy in the 4 weeks before the first planned dose of study medication, or had any active second malignancy within the previous 5 years, excluding basal cell carcinoma and squamous cell carcinoma of the skin, or cervical carcinoma *in situ*. Other exclusion criteria included: brain metastasis; clinically significant hypertension; acute or chronic bleeding requiring treatment within 3 months of the study start; functional iron deficiency (transferrin saturation < 20% and serum ferritin < 100 ng/mL); grade 3/4 thrombocytopenia (platelet count < 50 × 10^9^/L) or thrombocytosis (platelet count > 500 × 10^9^/L); creatinine > 2.5 mg/dL; C-reactive protein (CRP) > 50 mg/L; known folic acid/B_12 _deficiency or hemolysis; history of seizure, acute infection or inflammatory disease; pregnancy or lactation. Patients were also excluded if they had been treated with ESA therapy in the 8 weeks before study drug administration and were known to have resistance to ESA administration.

### Study design

This was a Phase II, randomized, open-label, multicenter, dose-finding study where patients were planned to receive C.E.R.A. SC injections over a 12-week treatment period. The design and conduct of the study complied with the principles of good clinical practice, in accordance with the Declaration of Helsinki. The study was approved by local ethics committees and informed written consent was obtained from all patients before enrollment.

#### C.E.R.A. administration and dose adjustment

Eligible patients were randomized to one of six treatment groups in two dose schedules: C.E.R.A. 0.7, 1.4, or 2.1 μg/kg QW or C.E.R.A. 2.1, 4.2, or 6.3 μg/kg Q3W. Patients received the first dose of study drug not more than 14 days after they were screened. The first dose of C.E.R.A. was given on day one of the cycle, before the administration of cyclic chemotherapy. Dose changes were made based upon the Hb levels on dosing day (within 24 hours). No dose increases of C.E.R.A. were allowed. If the Hb level increased by > 2 g/dL within 2 weeks, or if the Hb was > 12 g/dL and ≤ 13 g/dL, the current dose was reduced by 50%. Instructions were given to the investigators to reduce the dose only once during the study, when these criteria were met. Treatment was withheld if the Hb level was > 13 g/dL until a value of ≤ 12 g/dL was achieved. Treatment was then resumed at 50% of the previous dose. If the Hb level was > 14 g/dL, patients were evaluated for appropriate clinical intervention (for example, phlebotomy or use of fluids if hydration was considered necessary).

#### Assessments

Hematology parameters, vital signs, and body weight were measured at screening and weekly thereafter. Blood samples for pharmacokinetic assessment and laboratory tests (including blood chemistry and iron parameters) were taken and assessed at baseline and at regular intervals thereafter. Electrocardiogram (ECG) readings and anti-C.E.R.A. antibodies were analyzed at weeks 4, 8, 13 (or end of the study), and at a follow-up visit 8 weeks after the last dose of C.E.R.A. Adverse events, iron supplementation, blood transfusions, and concomitant medications were documented throughout the course of the study.

### Endpoints

#### Efficacy

The primary efficacy endpoint was the average Hb level between baseline and end of initial treatment, defined as the last Hb measurement before a dose reduction or transfusion, or the value at week 13, whichever came first. Secondary endpoints included the following parameters: the Hb response, which was defined as an increase in Hb of ≥ 2 g/dL from baseline on two consecutive measurements within a 10-day interval at any time during the study, with no blood transfusion in the previous 28 days; the hematopoietic response, which was defined as a Hb response or achievement of Hb ≥ 12 g/dL at any time during the study, with no blood transfusion in the previous 28 days. Changes in Hb, hematocrit, and reticulocyte counts were assessed over time, together with the requirement for any blood transfusions.

Two additional analyses not included in the study protocol were also performed. First, the average change in Hb level was assessed from baseline during week 5 to the end of study (week 13 or last assessment). In the event of blood transfusion, the last Hb value in the preceding 2 days was carried forward for the next 28 days. Second, responder categorization was performed as follows: 'early responders', defined as patients with a ≥ 1 g/dL increase in Hb from baseline during the first 22 days, with no concomitant blood transfusion during the study; 'additional responders', defined as patients who were not early responders but achieved the target therapeutic range of 11–13 g/dL during the study without blood transfusion; 'non-responders', defined as patients who did not qualify as 'early responders' or 'additional responders'.

#### Pharmacokinetics

Serum concentrations of C.E.R.A. were measured using an enzyme-linked immunosorbent assay (ELISA) with a limit of quantification of 150 pg/mL and used to determine the maximum serum concentration (C_max_) and time to maximum serum concentration (T_max_). The t_1/2 _was estimated for ln(2)/k, where the rate constant of elimination (k) was determined by linear regression on the logarithm of the serum concentration versus time data in the post-distribution phase. The area under the concentration-time curve (AUC) following C.E.R.A. administration, from pre-dose on day 22 until the last sampling time at which the concentration was measurable (day 29 for the QW group and day 43 for the Q3W group), was estimated by the linear trapezoidal rule.

#### Safety

Safety endpoints included adverse events, clinical laboratory tests, vital signs, body weight, physical examination, and ECGs. The intensities of adverse events and laboratory values were graded according to the National Cancer Institute/National Institutes of Health Common Toxicity Criteria.

### Statistical analyses

A sample size of 210 patients (35 per treatment group) was calculated to have 90% power in rejecting the overall F-test of no difference among the three treatment groups in each dose schedule group in the primary efficacy variable at the alpha level of 0.025 (2-sided). The 90% power and 0.025 significance level were chosen to address the multiple testing of the two-dosing schedule. This sample size included an anticipated dropout rate of 10%.

The efficacy of study medications was primarily evaluated for the intent-to-treat (ITT) population, which included patients who were randomized and received at least one dose of study medication. A per-protocol (PP) analysis was also performed, which included patients who met the inclusion criteria, did not receive blood transfusions during the study, and had no major protocol violations that are known to affect response to ESAs, such as blood transfusions in the 4-week period before first planned dose of C.E.R.A., functional iron deficiency (serum ferritin < 100 ng/mL and transferrin saturation < 20%), known hemolysis or acute infection or inflammatory disease (CRP > 50 mg/L). Patients with blood transfusions during the study were not considered protocol violations, but they were excluded from the PP analysis to avoid the compounding effect of transfusions on efficacy variables, such as change in Hb levels. The safety population included all randomized patients who received at least one dose of study medication.

The primary efficacy variable was the average Hb level during the period from baseline until the end of initial treatment (defined as last observed value before a dose change or blood transfusion) and was based on AUC using linear trapezoidal rule). This AUC approach was chosen in consideration of the commonly observed large variability in Hb measurements, since the cumulative effects on Hb level are assessed rather than Hb changes at one or two time points, enabling robust evaluation of biological activity of the treatment based on the benefits over the entire course of therapy.  Average Hb level from baseline to end of initial treatment was analyzed using an analysis of covariance (ANCOVA) model, with treatment group as a fixed factor and baseline Hb as a covariate. The average Hb change from baseline during week 5 until the end of study (week 13 or last available assessment) was also analyzed by ANCOVA, as for the primary efficacy variable. Cumulative response rates over time were estimated by Kaplan–Meier methods. All data, including Hb values for each assessment visit and pharmacokinetic parameters, were summarized using descriptive statistics.

## Results

### Patient disposition

**Figure 1:**
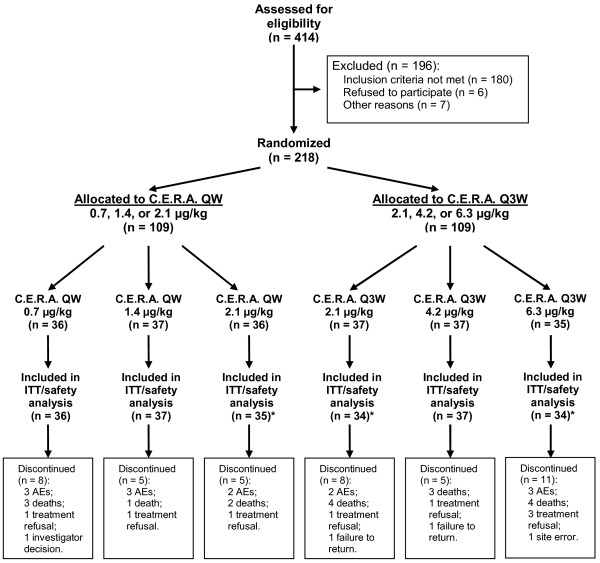
**Patient flowchart**. AEs: adverse events. C.E.R.A.: Continuous Erythropoietin Receptor Activator. ITT: intent to treat. QW: once weekly. Q3W: once ever 3 weeks. *One patient in the C.E.R.A. 2.1 μg/kg QW group, three patients in the 2.1 μg/kg Q3W group and one patient in the 6.3 μg/kg Q3W group were excluded from the ITT and safety analyses because they did not receive any study drug.

A total of 218 patients with NSCLC were enrolled into the study from 61 centers in North America, Central America, Europe, Asia, and Australia. Of these, 109 were randomized to one of the QW doses of C.E.R.A.; 36 to the 0.7 μg/kg group, 37 to the 1.4 μg/kg group, and 36 to the 2.1 μg/kg group. The other 109 patients were randomized to Q3W doses: 37 to the 2.1 μg/kg group, 37 to the 4.2 μg/kg group, and 35 to the 6.3 μg/kg group. The flow of patients through the study is shown in Figure 1. One patient in the QW group (2.1 μg/kg subgroup) and four patients in the Q3W group (three in the 2.1 μg/kg subgroup and one in the 6.3 μg/kg subgroup) did not receive any study drug after randomization and were not included in the ITT, PP, and safety analysis populations. The mean duration on study was similar across the six dose groups (74–81 days).

In the QW group, 18 patients were withdrawn from the study for the following reasons: adverse events (eight patients), death (six patients), refused treatment (three patients), and withdrawal at the discretion of the investigator (one patient). In the Q3W group, 24 patients were withdrawn for similar reasons: death (11 patients), adverse events (five patients), refused treatment or failed to return (seven patients), and a site error (one patient, who was started on third-line treatment [gefitinib] and the investigator thought the patient had to be withdrawn). Figure 1 gives the reasons for withdrawal by treatment group.

In the QW and Q3W groups, five and three further deaths, respectively, occurred after study completion. All the deaths were considered by the investigator to be unrelated to study medication. Overall, 44 and 36 patients in the QW and Q3W groups, respectively, were excluded from the PP population, the most common reason being the need for blood transfusion during treatment in 39 (89%) and 28 (78%) patients, respectively (Table 1).

**Table 1:** Analysis populations and reasons for exclusion from the per-protocol population.

	**C.E.R.A. dose group (μg/kg QW)**			**C.E.R.A. dose group (μg/kg Q3W)**		
	**0.7**	**1.4**	**2.1**	**2.1**	**4.2**	**6.3**
Number of patients randomized	36	37	36	37	37	35
Number of patients in ITT/safety population*	36	37	35	34	37	34
Number of patients in PP population	22	23	20	21	27	25
Number of randomized patients excluded from PP population	14	14	16	16	10	10
Reasons for exclusion from PP population**						
Blood transfusion during study period	13	12	14	16	7	5
Acute infection/inflammatory disease (CRP > 50 mg/L)	1	4	1	2	3	3
Received no study medication	0	0	1	3	0	1
Blood transfusion during 4-week period before study entry	0	0	0	3	0	0
Inclusion criteria not met	0	0	0	0	0	1

C.E.R.A.: Continuous Erythropoietin Receptor Activator.

CRP: C-reactive protein.

ITT: intent-to-treat.

PP: per-protocol.

QW: once weekly.

Q3W: once every 3 weeks.

*One patient in the C.E.R.A. 2.1 μg/kg QW group, three patients in the 2.1 μg/kg Q3W group and one patient in the 6.3 μg/kg Q3W group were excluded from the ITT and safety analyses because they did not receive any study drug.

**Patients may have had more than one reason for being excluded from the PP population.

### Baseline characteristics

The demographics and clinical characteristics at baseline were comparable among the QW and Q3W treatment groups (Table 2). The majority of patients had Stage IV NSCLC and had received chemotherapy before the start of the study. Approximately one-fifth to one-third of patients in each dose group had undergone surgery and/or radiotherapy. The median Hb level at baseline was similar among treatment groups, ranging from 10.1 g/dL to 10.6 g/dL (Table 2).

**Table 2:** Summary of patients' baseline characteristics (median values, except where indicated): safety population.

	**C.E.R.A. dose group (μg/kg QW)**			**C.E.R.A. dose group (μg/kg Q3W)**		
	**0.7**	**1.4**	**2.1**	**2.1**	**4.2**	**6.3**
Number of patients randomized	36	37	36	37	37	35
Sex, n, (%)						
Male	23 (64)	22 (59)	26 (72)	22 (59)	20 (54)	28 (80)
Female	13 (36)	15 (41)	10 (28)	15 (41)	17 (46)	7 (20)
Age, y (range)	66 (52–80)	65 (41–83)	66 (50–83)	63 (41–91)	63 (24–84)	64 (33–81)
Body weight, kg (range)	71 (41–127)	70 (39–102)	71 (41–91)	66 (36–90)	66 (39–89)	72 (47–100)
Disease stage, n, (%)						
IIIB	9 (25)	10 (27)	9 (26)	13 (38)	11 (30)	4 (12)
IV	27 (75)	27 (73)	26 (74)	21 (62)	26 (70)	30 (88)
Previous treatment before study entry, n, (%)						
Chemotherapy	32 (89)	35 (95)	30 (86)	33 (97)	34 (92)	31 (91)
Surgery	13 (36)	10 (27)	7 (20)	8 (24)	11 (30)	9 (26)
Radiotherapy	10 (28)	10 (27)	6 (17)	8 (24)	14 (38)	10 (29)
ESA	0	2 (5)	1 (3)	0	1 (3)	2 (6)
Hb level, g/dL (range)	10.2 (7.1–13.9)	10.2 (8.1–12.8)	10.3 (3.5–12.7)	10.4 (6.8–13.3)	10.1 (8.0–12.9)	10.6 (8.0–14.0)
Serum ferritin level, ng/mL (range)	350 (55–2188)	439 (48–1231)	285 (65–1403)	502 (92–1718)	472 (90–1225)	507 (79–1561)

C.E.R.A.: Continuous Erythropoietin Receptor Activator.

ESA: erythropoiesis-stimulating agent.

Hb: hemoglobin.

QW: once weekly.

Q3W: once every 3 weeks.

All patients received chemotherapy during the study, which was most commonly platinum based (carboplatin and cisplatin; 83%, 59%, and 71% in the 0.7, 1.4, and 2.1 μg/kg QW groups; 91%, 68%, and 65% in the 2.1, 4.2, and 6.3 μg/kg Q3W groups, respectively). Other most common chemotherapies included gemcitabine hydrochloride (43%–53% in the QW group and 41%–53% in the Q3W group) and taxanes (28%–46% in the QW group and 30%–44% in the Q3W group).

Overall, 97 patients received iron therapy and of these most (87 patients [90%]) received oral iron supplementation. The proportion of patients receiving intravenous iron supplementation was similar across all six C.E.R.A. groups. The percentage of patients receiving concomitant oral iron supplements was somewhat higher in patients who received C.E.R.A. Q3W (46% to 53%) than in patients treated QW (26% to 36%).

### Efficacy

Except where stated, all efficacy analyses were conducted for the ITT population. For the primary endpoint, the mean Hb change from baseline during the initial treatment period was for the QW group: -0.36 (95% confidence interval [CI]: -0.66 to -0.07), -0.19 (95% CI: -0.48 to +0.10), and -0.06 g/dL (95% CI: -0.36 to +0.24) in the 0.7, 1.4, and 2.1 μg/kg groups, respectively. For the Q3W group, the mean Hb changes were -0.23 (95% CI: -0.54 to +0.08), -0.07 (95% CI: -0.37 to +0.23), and 0.28 g/dL (95% CI: -0.03 to +0.59) in the 2.1, 4.2, and 6.3 μg/kg groups, respectively. Similar inclinations were observed in the PP population (mean increases -0.21, -0.08, 0.31 g/dL for the QW dose groups and 0.07, 0.23, 0.42 g/dL for the Q3W dose groups, respectively).

As Hb levels declined during the first few weeks of C.E.R.A. therapy in patients receiving concomitant chemotherapy, an additional analysis was performed to determine the average Hb changes from baseline during weeks 5 and 13 (or last assessment). Dose-dependent increases in Hb were observed in the QW and Q3W groups, with an increase of 0.66 g/dL in the 6.3 μg/kg Q3W group (Table 3).

**Table 3:** Average hemoglobin changes from baseline during weeks 5–13 (or last assessment): intent-to-treat population.

**C.E.R.A. dose group, μg/kg**	**n**	Least squares mean (95% CI)* Hb change (g/dL)
**QW**		
0.7	31	-0.24 (-0.79 to +0.31)
1.4	35	0.02 (-0.49 to +0.54)
2.1	34	0.40 (-0.12 to +0.93)

**Q3W**		
2.1	31	-0.16 (-0.75 to +0.42)
4.2	36	0.19 (-0.35 to +0.73)
6.3	33	0.66 (0.09 to +1.22)

C.E.R.A.: Continuous Erythropoietin Receptor Activator.

Hb: hemoglobin.

QW: once weekly.

Q3W: once every 3 weeks.

*Derived from analysis of covariance including dose group (QW and Q3W separately) as a fixed factor and the baseline value as a covariate in the model.

CI: confidence interval.

Hb response (defined as two consecutive, within 10-day increases from baseline Hb by ≥ 2 g/dL not related to transfusion) was reported for 8%, 22%, and 34% of patients in the 0.7, 1.4, and 2.1 μg/kg QW groups, respectively, and in 12%, 24%, and 26% of patients in the 2.1, 4.2, and 6.3 μg/kg Q3W groups, respectively. The proportion of patients achieving a hematopoietic response (Hb response or Hb ≥ 12 g/dL during the study) increased with increasing C.E.R.A. dose in both groups; 31%, 43%, and 63% of patients in the 0.7, 1.4, and 2.1 μg/kg QW groups, respectively, and 35%, 51%, and 62% of patients in the 2.1, 4.2, and 6.3 μg/kg Q3W groups, respectively.

**Figure 2:**
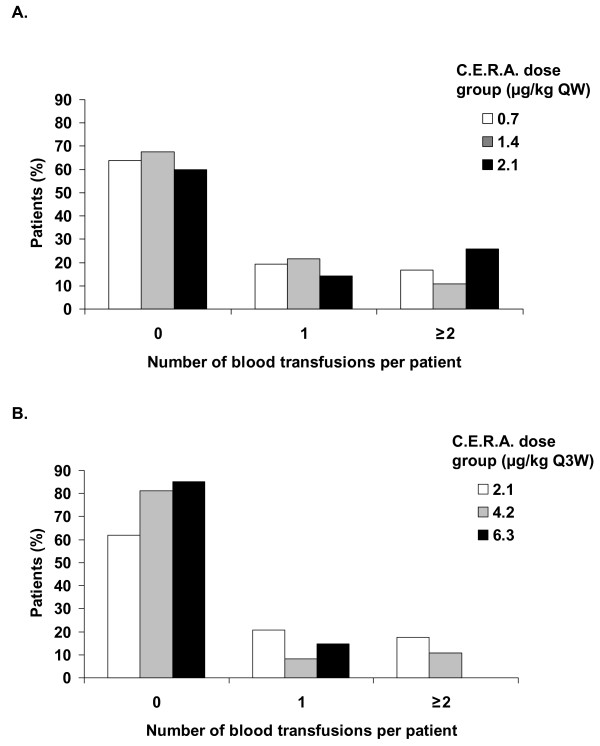
**Frequency of blood transfusions per patient**. (A) Group administered C.E.R.A. once weekly. (B) Group administered C.E.R.A. once every 3 weeks. Intent-to-treat population. C.E.R.A.: Continuous Erythropoietin Receptor Activator. QW: once weekly. Q3W: once every three weeks.

The majority of patients did not require blood transfusion during the study: 64%, 68%, and 60% of patients in the 0.7, 1.4, and 2.1 μg/kg QW groups, respectively, and 62%, 81%, and 85% of patients in the 2.1, 4.2, and 6.3 μg/kg Q3W groups, respectively, remained transfusion-free (Figure 2). In the 6.3 μg/kg Q3W group, no patient received more than one blood transfusion.

**Figure 3:**
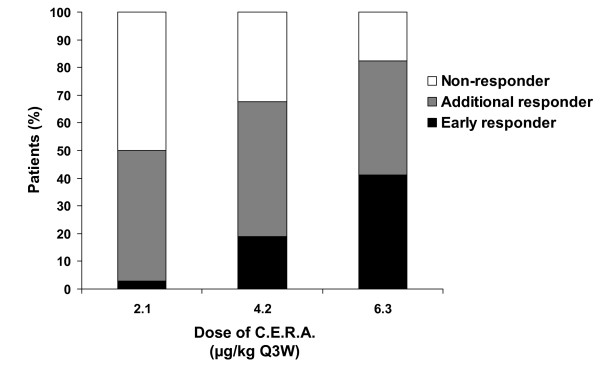
**Proportions of early, additional, and non-responders in group administered C.E.R.A. once every 3 weeks**. Intent-to-treat population. 'Early responders': patients with a ≥ 1 g/dL increase in hemoglobin (Hb) from baseline during the first 22 days, with no concomitant blood transfusion during the study. 'Additional responders': patients who were not early responders but achieved the target therapeutic range of 11–13 g/dL during the study without blood transfusion. 'Non-responders': patients who did not qualify as early responders or additional responders. C.E.R.A.: Continuous Erythropoietin Receptor Activator. Q3W: once every three weeks.

For the responder categorization analysis carried out on the Q3W group, a dose-dependent effect was seen in the proportion of 'early responders' and 'non-responders' (Figure 3). The proportion of 'additional responders' was similar in the 2.1 and 4.2 μg/kg groups and lowest in the 6.3 μg/kg group. The magnitude of mean Hb increase during weeks 5–13 (or last assessment) was higher in patients with an 'early response' than in those who had 'no response' regardless of the C.E.R.A. dose (-0.2 versus -1.1 g/dL in the 2.1 μg/kg group; 2.4 versus -1.5 g/dL in the 4.2 μg/kg group; 1.7 versus -1.2 g/dL in the 6.3 μg/kg group). However, these differences should be viewed with caution, as the numbers of patients in each group were small, particularly in the 2.1 μg/kg group (one patient with 'early response', 14 with 'no response').

### Pharmacokinetics

**Figure 4:**
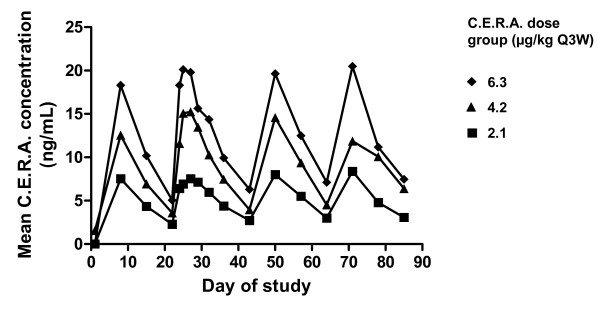
**Mean serum concentration-time profiles for C.E.R.A. after subcutaneous injection once every 3 week**s. C.E.R.A.: Continuous Erythropoietin Receptor Activator. Q3W: once every three weeks.

Pharmacokinetic parameters following SC administration of C.E.R.A. QW and Q3W are summarized in Table 4. Median T_max _values were 70–74 hours (3 days) in the QW group and 82–120 hours (3–5 days) in the Q3W group. Mean C_max _and AUC_22–29 days _or AUC_22–43 days _values increased with increasing C.E.R.A. dose in both the QW and Q3W groups. In the Q3W group, due to low clearance (median values 34.7–49.4 mL/h/kg), mean t_1/2 _values were long, ranging from 6.5–7.8 days. The C.E.R.A. half-life could not be determined in the QW group because of low patient numbers (n = 4, 2, 3 in the 0.7, 1.4, and 2.1 μg/kg groups, respectively). The mean C.E.R.A. serum concentrations over time in the Q3W group are shown in Figure 4.

**Table 4:** Pharmacokinetic values for C.E.R.A. after subcutaneous injection. Results are given as mean ± SD, apart from T_max _and CL/F, which are given as median values.

**C.E.R.A. dose group, μg/kg (n)**	**C_max _(ng/mL)**	**T_max _(h)**	AUC (ng • h/mL)*	**t_1/2 _(h)**	**CL/F (mL/h/kg)**
**QW**					
0.7 (14)	6.7 ± 2.5	71	931 ± 358	171 ± 69^†^	25.3
1.4 (16)	10.6 ± 4.1	74	1355 ± 548^‡^	NC**	25.1
2.1 (15)	20.2 ± 8.7	70	2709 ± 1193	212 ± 200^†^	23.4

**Q3W**					
2.1 (18)	8.2 ± 3.8	120	2750 ± 1318	157 ± 38^†^	34.7
4.2 (18)	16.6 ± 9.5	82	4651 ± 2569^‡^	174 ± 44^†^	42.8
6.3 (17)	20.9 ± 12.5	120	6547 ± 3957^‡^	186 ± 78^†^	49.4

AUC: area under the serum concentration-time curve.

C.E.R.A.: Continuous Erythropoietin Receptor Activator.

CL: clearance.

C_max_: maximum serum concentration.

F: bioavailability.

QW: once weekly.

Q3W: once every 3 weeks.

SD: standard deviation.

T_max_: time to C_max_.

t_1/2_: terminal half-life.

*AUC_22–29 days _for QW group and AUC_22–43 days _for Q3W group.

**NC, not calculated (n = 2).

^†^n = 4 and 3 in 0.7 and 2.1 QW groups, respectively, and n = 11, 15, 14 in 2.1, 4.2, and 6.3 Q3W groups, respectively.

^‡^n = 15, 16, and 14 for 1.4 QW. 4.2 Q3W, and 6.3 Q3W groups, respectively.

### Tolerability and safety

C.E.R.A. dose modification (dose reduced or withheld) was more frequent in the QW group compared with the Q3W group, probably because of the larger number of opportunities to change dose in the QW group. There did not appear to be a dose-related trend. In the QW group, six (17%), 14 (38%), and 15 (43%) patients in the 0.7, 1.4, and 2.1 μg/kg groups, respectively, had C.E.R.A. dose modifications. In the Q3W group, five (15%), 11 (30%), and nine (27%) patients in the 2.1, 4.2, and 6.3 μg/kg groups, respectively, had dose modifications.

C.E.R.A. doses of 0.7, 1.4, or 2.1 μg/kg given QW and 2.1, 4.2, or 6.3 μg/kg given Q3W were generally well tolerated. The QW and Q3W groups showed similar tolerability profiles (Table 5). The proportion of patients reporting adverse events was similar across all treatment groups. Events were reported by 89%, 97%, and 94% in the 0.7, 1.4, and 2.1 μg/kg QW groups, respectively, and in 94%, 95%, and 97% of patients in the 2.1, 4.2, and 6.3 μg/kg Q3W groups, respectively. The most frequently reported adverse events were nausea, fatigue, anorexia, vomiting, and neutropenia (Table 5), and were most likely secondary to chemotherapy. There did not appear to be a dose-related trend in the incidence of events.

**Table 5:** Most frequently reported adverse events (in ≥ 5% of patients in any treatment group). Patients administered C.E.R.A. QW or Q3W.

	**C.E.R.A. dose group (μg/kg QW)**			**C.E.R.A. dose group (μg/kg Q3W)**		
**Adverse event, n (%)**	**0.7 (n = 36)**	**1.4 (n = 37)**	**2.1 (n = 35)**	**2.1 (n = 34)**	**4.2 (n = 37)**	**6.3 (n = 34)**
Nausea	12 (33)	6 (16)	11 (31)	11 (32)	7 (19)	4 (12)
Fatigue	11 (31)	8 (22)	7 (20)	6 (18)	10 (27)	7 (21)
Anorexia	7 (19)	7 (19)	7 (20)	6 (18)	7 (19)	6 (18)
Vomiting	8 (22)	7 (19)	9 (26)	6 (18)	2 (5)	6 (18)
Neutropenia	9 (25)	3 (8)	6 (17)	8 (24)	7 (19)	4 (12)
Asthenia	5 (14)	5 (14)	5 (14)	6 (18)	5 (14)	4 (12)
Cough	8 (22)	3 (8)	3 (9)	5 (15)	4 (11)	4 (12)
Diarrhea	6 (17)	5 (14)	4 (11)	3 (9)	6 (16)	3 (9)

C.E.R.A.: Continuous Erythropoietin Receptor Activator.

QW: once weekly.

Q3W: once every 3 weeks.

There were 13 and 14 deaths in the QW and Q3W dose groups, respectively, none of which was considered by the investigator to be related to the study medication. Six and four patients in the QW (5.6%) and Q3W dose group (3.8%), respectively, had adverse events that were considered by the investigator to be related to study treatment. In the 0.7 μg/kg QW group, one patient had four adverse events considered related to treatment (back pain, anemia, neutropenia, and hypersensitivity), one patient experienced injection site pain, one patient had pyrexia, and one patient had bone pain. In the 1.4 and 2.1 μg/kg QW groups, one patient in each group had thrombophlebitis. In the 2.1 μg/kg Q3W group, one patient had injection site discomfort; in the 4.2 μg/kg Q3W group, one patient experienced flushing, hypertension, and headache, and another patient had bruising at the injection site; in the 6.3 μg/kg Q3W group, one patient was reported with atrial fibrillation. Of these patients, only the patient with atrial fibrillation withdrew from the study due to the adverse event. The patient was hospitalized and treated with digoxin 0.25 mg. Normal sinus rhythm was restored soon after admission, although second degree atrioventricular heart block was recorded. Four other adverse events led to premature withdrawal from the study in the Q3W dose group (two in the 2.1 μg/kg group and two in the 6.3 μg/kg group). The reasons for withdrawal were brain metastasis (one patient) and NSCLC progression (three patients). In the QW group, eight adverse events led to premature withdrawal (three in the 0.7 μg/kg group, three in the 1.4 μg/kg group, and two in the 2.1 μg/kg group). The reasons for withdrawal were NSCLC progression (five patients), renal failure (one patient), worsening of anemia (one patient), and hemoptysis (one patient).

Three patients in the QW group (2.8%; two in the 1.4 μg/kg group and one in the 2.1 μg/kg group) and two patients in the Q3W group (1.9%; both in the 4.2 μg/kg group) had thrombovascular events (TVEs), including thrombotic events. In the Q3W group, myocardial infarction was reported in one patient and cerebrovascular accident in another. None of the TVEs were considered by the investigator to be related to treatment with C.E.R.A. No TVEs were reported in the 6.3 μg/kg Q3W group.

There were no clinically significant changes from baseline in laboratory values and vital signs during the study in all dose groups, or dose-dependent relationships for increasing blood pressure. In addition, no anti-C.E.R.A. antibodies were detected.

## Discussion

Treatment with ESAs is now the standard of care for chemotherapy-induced anemia, resulting in increased Hb levels, reduced transfusion requirements, and improved QoL [18-22]. However, new treatments that allow less frequent administration and coordination of administration with chemotherapy regimens, and with improved early and sustained Hb response, would further improve the clinical profile of ESAs. C.E.R.A. is the first continuous erythropoietin receptor activator developed for the control of anemia in patients with cancer. In preclinical studies and healthy volunteers, C.E.R.A. was shown to have a prolonged serum half-life, suggesting that it may be administered at extended intervals when compared with ESAs [28,29]. A small study in patients with multiple myeloma demonstrated dose-dependent increases in maximum C.E.R.A. serum concentration and Hb levels over a 6-week treatment period using Q3W administration and doses up to 8 μg/kg [27]. In addition, the proportion of patients with a Hb response in patients treated with C.E.R.A. 3.5–8.0 μg/kg (50.0%–62.5%) compared favorably with values obtained in studies of epoetin beta and darbepoetin alfa given QW in cancer patients receiving myelosuppressive chemotherapy [19,31]. The present study examined the efficacy, safety, and pharmacokinetic profile of C.E.R.A. in anemic patients with Stage IIIB or IV NSCLC receiving myelosuppressive chemotherapy. The dose range chosen was based on the doses shown to be effective in the multiple myeloma study [27]. In this study, patients with NSCLC receiving chemotherapy were administered one of six doses of C.E.R.A. (0.7–2.1 μg/kg QW or 2.1–6.3 μg/kg Q3W) by SC injection.

The results demonstrated that C.E.R.A. treatment led to dose-related improvements in patients' anemia. Increases in mean Hb level and Hb response were observed with increasing doses of C.E.R.A. administered Q3W and QW. Dose-dependent increases in Hb were also observed when the change from baseline during week 5 to week 13 was assessed; but it is important to achieve more substantial erythropoietic benefits, which could be observed with higher doses of C.E.R.A. It is also interesting to note that, although the study was not powered to compare dose schedules, there was an inclination for higher increases in mean Hb level overall and during week 5 to week 13 when the same dose over time was given Q3W rather than QW.

The majority of patients in the 4.2 and 6.3 μg/kg Q3W groups (51% and 62%, respectively) and 2.1 μg/kg QW group (63%) achieved a hematopoietic response. In addition, in the Q3W group, the proportion of 'early responder' patients increased with increasing C.E.R.A. dose, reaching 41% with the highest dose. Adding together the proportions of 'early responders' and 'additional responders', the total response in the 6.3 μg/kg group was 82%. Conversely, the proportion of patients who did not respond to C.E.R.A. treatment decreased with increasing C.E.R.A. dose. There was also an indication that early response was predictive of later Hb increases. The magnitude of mean Hb increase during weeks 5–13 was higher in 'early responders' than in patients who did not show early response.

The majority of patients in all six treatment groups did not require blood transfusion during the study. There was a trend for a greater transfusion-saving effect with C.E.R.A. administered Q3W compared with QW treatment; 60% of patients in the 2.1 μg/kg QW group did not require transfusion in comparison with 85% of patients administered the same weekly dose Q3W (6.3 μg/kg Q3W). Also, in the 6.3 μg/kg Q3W group, of the 15% of patients that required blood transfusion, all received only one transfusion during the study. This suggests a positive drug effect, as previous studies suggested a very high rate (up to 59%) of transfusion use in patients with lung cancer receiving platinum therapy without the support of an ESA [32,33].

The major limitations of the current study design included the lack of a placebo or active control group in the study, the limited range of C.E.R.A. doses, and the lack of dose increase in the event of an inadequate Hb response. Most other studies of ESAs in patients with lung cancer allowed dose increases in patients who did not respond [20,34-36]. Despite this, the Hb and hematopoietic response rates from this study compare well with those from studies of ESA treatment of anemia in patients with lung cancer receiving chemotherapy. In a study of darbepoetin alfa QW treatment, the proportion of patients with a hematopoietic response (defined as an increase in Hb of ≥ 2.0 g/dL or Hb ≥ 12 g/dL in the absence of transfusion in the previous 28 days) was 66% [20]. This response rate was similar to the 62% hematopoietic response rate achieved in the C.E.R.A. 6.3 μg/kg Q3W group in the present study. However, a more stringent definition of response was applied in the current study (confirmed on two consecutive measurements in this study versus a single measurement in the darbepoetin alfa study). Also, nearly half of patients (43%) in the darbepoetin alfa study required dose doubling to 4.5 μg/kg QW because of inadequate response to 2.25 μg/kg QW [20,37]. Therefore, it is reasonable to hypothesize that response rates in the present study could have been even higher if dose increases had been permitted.

Despite the presence of some similarities with other studies, one has to be careful when comparing the results of the present study with those of some previous trials of ESA treatment in lung cancer. Most previous ESA studies included a mixture of patients with small cell lung cancer and NSCLC [20,34-36,38-40] in contrast to this study which included only Stage IIIB or IV NSCLC patients. Other factors compromising between-study comparisons include differences in patient populations in terms of disease stages and chemotherapy intensity. In terms of disease stage, a study of darbepoetin alfa 200 μg every 2 weeks and epoetin alfa 40,000 IU QW examined the hematopoietic response in patients with breast, gynecologic, or Stage IIIB/IV NSCLC [36]. The mean change from baseline Hb at the end of treatment (up to 16 weeks) was lowest in patients with advanced NSCLC: 0.6 and 1.3 g/dL in the darbepoetin alfa and epoetin alfa groups, respectively, in comparison with increases of 1.7–1.9 g/dL or 1.3 g/dL in patients with breast cancer or gynecologic cancer. The late disease stage was thought to have contributed to the poorer responses in the NSCLC patients [36]. Also, the intensity of chemotherapy varied in previous studies of patients with Stage III/IV NSCLC [20,35,36,39]. In the present study, most patients had received chemotherapy before the study and all received chemotherapy during the study, most commonly platinum based (65% to 91%). The use of platinum compounds has been shown to have a negative impact on Hb levels in patients with lung cancer [5].

The pharmacokinetic profile of C.E.R.A., including a prolonged half-life and low clearance, is different to that of epoetin alfa and darbepoetin alfa [26]. The half-life of C.E.R.A. was considerably longer than that of the ESAs. Epoetin alfa has a half-life of approximately 40–44 hours in cancer patients [41,42] and approximately 19 hours in healthy volunteers [43]. Darbepoetin alfa has a half-life of 39–74 hours in cancer patients [44,45] and 49 hours in patients with kidney disease [46]. The t_1/2 _(mean 157–186 hours) of C.E.R.A. reported in this study was similar to the values observed in patients with multiple myeloma [27], healthy volunteers [29], and renal patients [47].

C.E.R.A. administered Q3W and QW with concomitant chemotherapy was generally well tolerated across all dose groups. The adverse events reported were mainly related to the effects of chemotherapy or the underlying disease. The incidences of fatigue, anorexia, and dyspnea were similar to those reported in the darbepoetin alfa trial of lung cancer patients [20], whereas the incidence of nausea, most likely related to chemotherapy, appeared to be lower in the C.E.R.A. study (12%–33% versus approximately 42%). On the other hand, in a retrospective analysis of three epoetin alfa lung cancer studies, the incidence of adverse events was lower than in the C.E.R.A. study [34]. These different findings may be explained by dose non-comparability and methodological differences between the separate studies, making direct comparisons difficult. TVEs were reported by three patients in the QW group (two in the 1.4 μg/kg dose group and one in the 2.1 μg/kg dose group) and two patients in the Q3W group (4.2 μg/kg dose group). There were no TVEs in the 6.3 μg/kg dose Q3W group. All TVEs were considered to be unrelated to C.E.R.A. by the investigator. This TVE incidence is similar to that reported with placebo treatment in meta-analyses of ESA studies [48,49].

## Conclusion

The Hb results in this study show that C.E.R.A. administered SC QW or Q3W has clinical activity in patients with Stage IIIB (pleural effusion) or IV NSCLC. There were dose-dependent and sustained increases in response related to Hb level with increasing C.E.R.A. dose levels on QW and Q3W dosing schedules. The proportion of patients with early responses also increased with increasing C.E.R.A. dose administered Q3W, reaching 41% in the 6.3 μg/kg group. There was an inclination for improved effectiveness of C.E.R.A. in terms of mean Hb level increases and transfusion use when the same dose over time was given Q3W as opposed to QW. In the 6.3 μg/kg Q3W group, most patients remained transfusion-free (85%) during the study, with no patients in the 6.3 μg/kg group requiring more than one transfusion. C.E.R.A. was generally well tolerated across all dose groups. These results suggest that C.E.R.A. Q3W has the potential to reduce the burden of anemia management for patients and healthcare providers by providing an effective alternative to transfusions in a treatment that can be synchronized with a patient's chemotherapy regimen.

Confirmation of the previously defined pharmacokinetic profile of C.E.R.A., and its good tolerability and safety profile in this study of patients with advanced NSCLC, also suggest that extended administration intervals are feasible in the clinic. However, further dose-finding studies that use higher doses and allow dose escalation are required to determine the optimal C.E.R.A. Q3W dose regimen in anemic cancer patients receiving chemotherapy. The results of other studies with C.E.R.A. in the treatment of chemotherapy-related anemia are awaited.

## Competing interests

VH, JG, CM, PM and RR have received honoraria from Hoffmann-La Roche. JEE is an employee of Hoffmann-La Roche Inc. and possesses shares in Hoffmann-La Roche.

## Authors' contributions

VH was Principle Investigator for the study described in the manuscript. VH, JG, PM, CM, and RR participated in designing and performing the research. JEE participated in the analysis and interpretation of data. All authors participated in drafting and revising the manuscript and all authors read and approved the final manuscript.
